# Evaluation of Flow-Induced Shear in a Porous Microfluidic Slide: CFD Analysis and Experimental Investigation

**DOI:** 10.3390/fluids10060160

**Published:** 2025-06-17

**Authors:** Manoela Neves, Gayathri Aparnasai Reddy, Anitha Niyingenera, Norah Delaney, Wilson S. Meng, Rana Zakerzadeh

**Affiliations:** 1 Department of Biomedical Engineering, School of Science and Engineering, Duquesne University, Pittsburgh, PA 15219, USA; 2 Division of Pharmaceutical Sciences, School of Pharmacy, Duquesne University, Pittsburgh, PA 15282, USA; 3 McCowan Institute for Regenerative Medicine, University of Pittsburgh, Pittsburgh, PA 15219, USA

**Keywords:** microfluidic devices, computational fluid dynamics (CFD), modeling and simulation, porous extracellular matrix, filtration velocity, shear stress

## Abstract

Microfluidic devices offer well-defined physical environments that are suitable for effective cell seeding and in vitro three-dimensional (3D) cell culture experiments. These platforms have been employed to model in vivo conditions for studying mechanical forces, cell–extracellular matrix (ECM) interactions, and to elucidate transport mechanisms in 3D tissue-like structures, such as tumor and lymph node organoids. Studies have shown that fluid flow behavior in microfluidic slides (μ-slides) directly influences shear stress, which has emerged as a key factor affecting cell proliferation and differentiation. This study investigates fluid flow in the porous channel of a μ-slide using computational fluid dynamics (CFD) techniques to analyze the impact of perfusion flow rate and porous properties on resulting shear stresses. The model of the μ-slide filled with a permeable biomaterial is considered. Porous media fluid flow in the channel is characterized by adding a momentum loss term to the standard Navier–Stokes equations, with a physiological range of permeability values. Numerical simulations are conducted to obtain data and contour plots of the filtration velocity and flow-induced shear stress distributions within the device channel. The filtration flow is subsequently measured by performing protein perfusions into the slide embedded with native human-derived ECM, while the flow rate is controlled using a syringe pump. The relationships between inlet flow rate and shear stress, as well as filtration flow and ECM permeability, are analyzed. The findings provide insights into the impact of shear stress, informing the optimization of perfusion conditions for studying tissues and cells under fluid flow.

## 1. Introduction

Prefabricated microfluidic slides (μ-slides) present channels with specific geometries to accommodate biological environments for three-dimensional (3D) cellular organization and proliferation, including seeding, sampling, and analysis [1]. The understanding of nutrient exchange and transport mechanisms in various 3D tissue-like structures known as organoids, particularly in recapitulating the human tumor microenvironment and lymph node-mimicking systems, can be advanced through microfluidic-based platforms [2]. Specifically, since most cells need signals from a 3D domain for relevant physiological behavior and response, these devices recreate a suitable environment to serve as a model for in vitro tissue engineering applications such as implantation and tissue repairing, stem cell behavior studies and organ developmental processes [3], as well as disease modeling and drug discovery screening, in controllable 3D forms [4].

Porous artificial extracellular matrix (ECM) scaffolds accommodate cell differentiation and tissue growth under physiologically relevant conditions [4]. For this purpose, biocompatible materials that support cell attachment and differentiation, while minimizing immune response and inflammation, are highly desirable. The μ-slide platforms have been used to configure biological mimetics for the study of mechanical forces and cell–ECM interactions, by providing controlled dynamic flow conditions for physical factors such as fluid shear stress [5]. Overall, 3D culture techniques and the application of a dynamic setting (e.g., shear stress) more accurately represent tissues’ nature and lead to more reliable outcomes than conventional two-dimensional static cell cultures.

Flow-induced shear stress in microfluidic devices is a crucial element steering biological responses in instances of monolayer cell cultures and 3D organoids [6], making it an important subject of investigation in biomedical research [7]. Previous literature has shown that the fluid flow behavior in these devices directly impacts shear stress, which has been observed as a point of interest regarding its effect on cellular morphology and alignment, cellular growth rate and differentiation [8], and gene expression [9,10]. Maintaining shear stress within the physiological range is fundamental for cellular functions and viability. When the shear stress values are below this range, cells can undergo apoptosis. Conversely, substantial values of shear forces can alter the cytoskeletal structure, and the cells can be washed away with the fluid flow.

Given the utility of microfluidic systems in studying cellular behavior and mechanisms in response to fluid dynamics parameters (such as shear stress, pressure, and flow rate), advanced computational fluid dynamics (CFD) modeling techniques are essential for accurately simulating these conditions in quantitative terms, necessary for optimization and selecting suitable conditions in these devices. The use of CFD approaches also offers an efficient method of conducting larger basic and applied research screenings of the physical features affecting biological processes and can provide high throughput and content to support in vitro and in vivo studies, as well as to access specific outputs. A comprehensive review by Huang et al. demonstrates the role of CFD in studying fluid dynamics and nutrient transport within these systems, as well as the need for sophisticated computational models to precisely forecast cellular functions [11].

As fluid flow processes have a direct effect on shear stress and, therefore, implications for cellular responses, several studies have investigated the derivations of these parameters using CFD. Lindner et al. [12] designed a complementary set of reusable culture chambers based on the conducted CFD simulations and demonstrated the high impact of surface shear stresses obtained by numerical methods on the differentiation and reorganization of intestinal epithelial cells. Rosa et al. [13] developed a microfluidic platform that allows the real-time study of flowing lymphocytes dynamically interacting with adherent dendritic cells and examined how different shear stress values within the channel, predicted by the computational model, form the intercellular dynamics in a continuous flow, as a way to mimic the natural principle of interaction that occurs in vivo. Delon et al. [14] assessed the effects of the fluid shear stress on cellular monolayer morphology and key characteristics in a Hele–Shaw microfluidic device, where computational simulation of the fluid flow provided a guideline on how to adjust the experimental parameters to apply specific ranges of shear forces and precisely map out the shear stress distribution within the device. Bakuova et al. [15] compared flow behavior and cell viability between various geometries of chambered microfluidic devices, highlighting the impact of design on optimizing fluid flow and subsequently regulating shear stress distributions. Pisapia et al. [16] investigated various fluid dynamic parameters within the μ-slide and demonstrated strategies to predict and control the effects of shear forces in the human vascular system to recapitulate the cell- and organ-specific microenvironments. Bissoyi et al. [17] conducted numerical modeling of flow in microchannels by varying inlet velocity and observed the effects on flow-induced shear stress distribution, mesenchymal stem cells viability, and metabolic activity. Moreover, through a CFD modeling approach, Bahmaee et al. [18] evaluated the impact of fluid flow and shear stresses on osteogenic cellular behavior, particularly differentiation and matrix formation.

While several previously published studies have incorporated CFD analysis and thus quantified the shear stress in microfluidic-based platforms by numerically solving the fluid flow equations, none of them addressed the estimation of shear forces in 3D matrices independent of cellular elements. Given the pivotal nature of shear stress in microfluidic devices, delineation of matrix vs. cell-induced effects is a significant need for the analysis of fluid transport in ECM-based porous structures. Few studies have evaluated various aspects of fluid flow in porous scaffolds using computer simulation techniques to characterize scaffold architecture [9], calculated its permeability [19], or modeled the transport of biomolecules [20]; however, to our knowledge, there is no previous research about the interaction of fluid flow and porous scaffolds for a commercially available microfluidic system. Examining fluidic metrics, such as filtration velocity, shear stress, fluid pressure, and the parameters influencing their alteration (i.e., the structure and porosity of the surrounding ECM) is fundamental in regulating numerous biological processes. Yet these parameters have not been explored specifically in systems with defined physical dimensions. Incorporating computational approaches for the simulation of fluid dynamics in this context is the focus of this work.

The objective of this research is to develop and verify a framework that utilizes CFD techniques to quantify fluid filtration and resultant flow-induced wall shear stress patterns in the porous channel of a commercially available μ-slide. The prefabricated channel is assumed to be filled with a porous biological material, which represents the characteristics of native human ECM. By performing numerical simulations, the predicted filtration velocity and shear stress values throughout the permeable region (i.e., the device channel) are evaluated for a physiological range of known ECM permeability coefficients and inlet flow rate conditions. Subsequently, the experiment utilizes the same setup for measuring the filtration velocity by performing protein perfusions into the slide embedded with a hydrogel component that mimics the natural environment, while the flow rate is controlled by a syringe pump. The estimated experimental value of fluid perfusion is compared with CFD results.

Quantification of shear stress facilitates the design of experiments aimed at creating artificial ECM scaffolds that support effective cell proliferation, differentiation, and tissue regeneration under physiologically relevant conditions. Moreover, understanding the relationship between filtration flow and ECM permeability is imperative for controlling shear stress in microfluidic devices used for 3D cellular organization. The findings provide a basis for predicting fluid dynamic metrics within native human ECM, thereby enabling a mechanistic understanding of perfusion in porous microfluidic devices and ECM mimetics, ultimately advancing the development and optimization of human organoids.

## 2. Mathematical Model and Methods

A mathematical-computational model of a μ-slide channel filled with porous material is developed to determine the fluid perfusion and resulting shear stresses. Fluid is perfused at defined rates as experimentally derived properties for culture media, and CFD simulations are performed to obtain data on the filtration velocity and wall shear stress induced by the fluid flow. The details of this procedure are provided in this section.

### 2.1. Problem Description

**Figure 1.**
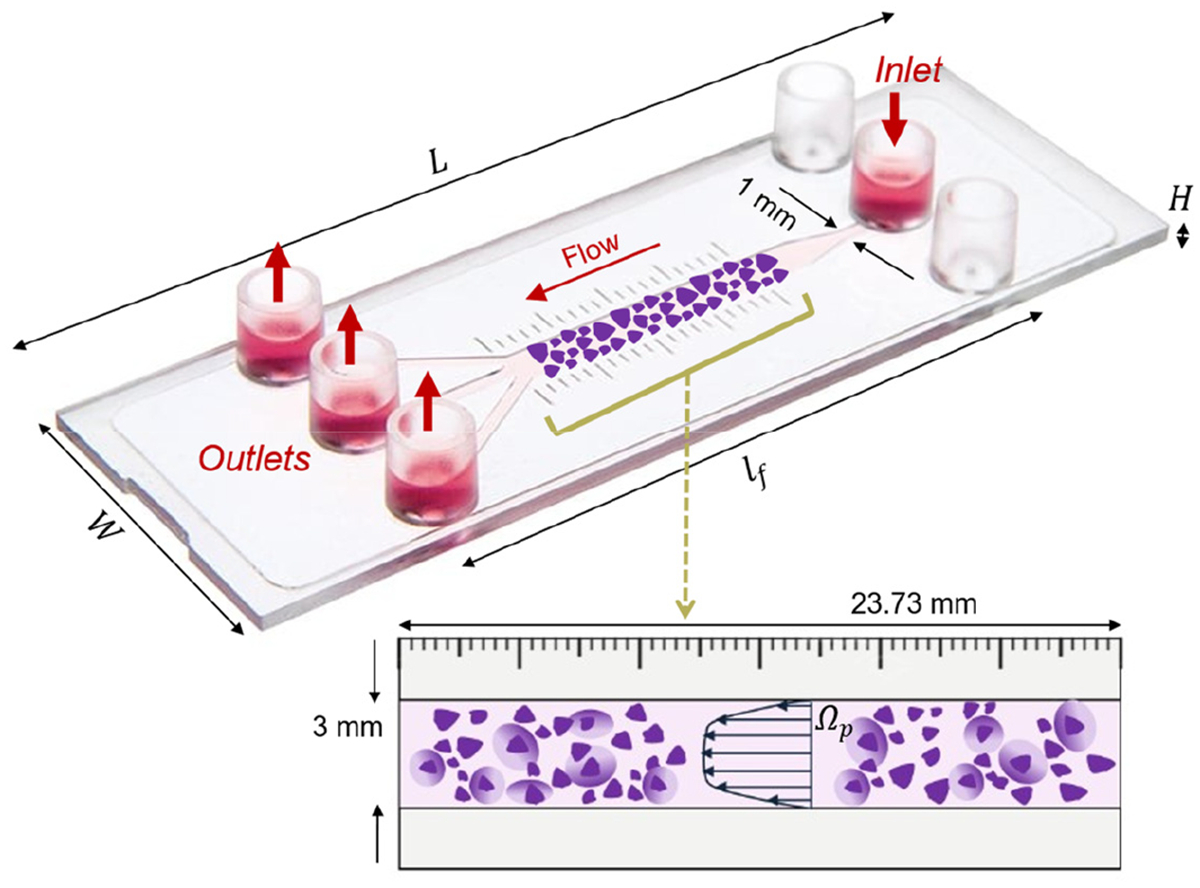
Representation of the three-dimensional ibidi^®^ μ-Slide III 3in1 microfluidic device geometrical features, with the schematic of the 3 mm channel (interrogation area, represented in purple) filled with a porous human ECM gel (Ω_*p*_). *l*_*f*_ and *H* indicate the length and height of the 3 mm channel. The fluid chamber measured 45 mm in length, with the 3 mm channel accounting for 23.73 mm of that. The fluid flow direction, one inlet and three outlets are indicated by red arrows.

The setup consists of the commercial microfluidic flow kit (μ-Slide III 3in1, ibidi GmbH, Gräfelfing, Germany), which is made of one rectangular channel that expands from a single 1 mm thin inlet to a thicker 3 mm main channel and splits up into three 1 mm smaller ones. The thick 3 mm single channel is the region of interest for this study. The device contains a millimeter scale bar next to the main channel, with defined 1 mm marks that allow for the quantification of movement through the channel. The thinner 1 mm channels can be used as an inlet or outlet interchangeably. The slide and its one-inlet and three-outlet configuration used for the setup of this study are displayed in Figure 1.

To generate a construct that mimics tissue physiology, we consider that the μ-slide chamber is filled with a porous human-derived ECM reconstituted in buffered saline, resulting in an estimated 90% porosity [21,22]. The channel is specifically designed for the cultivation of tissues and cells under fluid flow and is conducive for optical imaging. The three-dimensional geometry of the considered platform is created using Autodesk Fusion 360 CAD modeling software based on the dimensions obtained from the product’s instruction sheet, listed in Table 1. Additionally, each of the inlet and outlet 1 mm channels is characterized by a length of 5.3 mm.

### 2.2. Flow in μ-Slide Channel: Governing Equations and Modeling Details

The governing equation for the fluid flow in the porous μ-slide channel is provided by (1), which has the structure of the Brinkman equation that combines viscous terms, such as in Stokes, with friction terms, such as in Darcy. Namely, the effects of porous media on macroscopic flow (such as pressure drop and turbulence intensity) are accounted for by adding an extra momentum loss term to the Navier–Stokes equations. This flow resistance term is composed of a viscous loss term represented by Darcy, represented by the first term on the right-hand side of Equation (1). Culture medium fluid in the microfluidic channel is assumed to behave as an incompressible, Newtonian, viscous fluid. Flow is also considered to be laminar and under steady conditions. Therefore, the momentum balance and the continuity equations read as follows:

(1) $$\nabla \cdot \left(\rho_{f}\left(v_{f}\times v_{f}\right)\right)-\nabla \cdot \left(\mu_{f}\left(\nabla v_{f}+{\left(\nabla v_{f}\right)}^{T}\right)\right)=-\frac{\mu_{f}}{K_{p}}v_{f}-\nabla p_{f}\;\text{in}\;\Omega_{p}\nabla \cdot v_{f}=0\;\text{in}\;\Omega_{p}$$

In (1), variable ***v***_*f*_ denotes the velocity field through the porous medium. This velocity is also referred to as filtration velocity, defined as the relative velocity between the fluid and solid phases, multiplied by the porosity, *γ*_*p*_. The fluid pressure inside the permeable domain is referred to by *p*_*f*_. The density and dynamic viscosity of the culture medium that perfuses through the μ-slide are denoted in Equation (1) by *ρ*_*f*_ and *μ*_*f*_, respectively. The fluid in the pores is estimated to have the properties of water, which is found to be an appropriate model for culture medium in CFD studies [23]. The corresponding values of material properties are provided in Table 1.

Furthermore, the parameter *K*_*p*_ stands for the permeability coefficient of the porous medium, which is a scalar quantity for the porous μ-slide channel. The permeability for the baseline case is set to *K*_*p*_ = 10^−13^ m^2^, which is similar to the measured value for ECM derived from human placentas [24]. Since the estimated permeability in the literature fluctuates widely for ECM-based scaffolds, to determine the effect of scaffold porous features, five sets of the permeability coefficients in the range of 10^−15^ to 10^−7^ m^2^ are considered with exponentially increasing values of *K*_*p*_ = 10^−15^, 10^−13^, 10^−12^, 10^−9^, and 10^−7^ m^2^, as shown in Table 2. The values for permeability in this range are determined to be comparable to those reported in the existing literature for ECM permeability of various human and animal tissues [25–27] and collagen-based scaffolds [28,29]. Additionally, in Table 2, the permeability value of infinity indicates the free flow case (*K*_*p*_ → ∞).

### 2.3. CFD Numerical Simulation Setup

**Figure 2.**
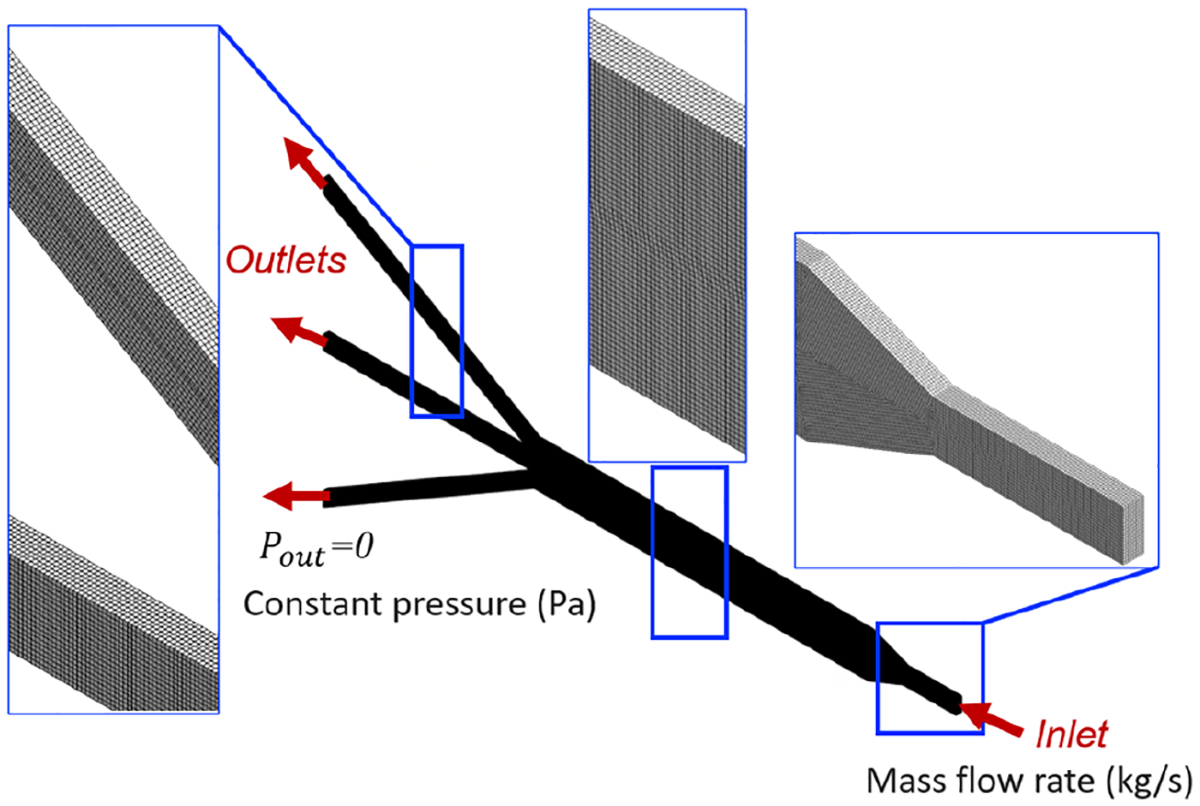
Domain discretization and boundary conditions. The zoomed-in inserts show the computational mesh.

By numerically solving the governing equations of the fluid flow in the μ-slide (Equation (1)), the computational model predicts the velocity and pressure fields within the microfluidic device, which are crucial for determining shear stresses. The details of the computer simulation process are elaborated in this section. The model, computational mesh, and definition of boundary conditions are illustrated in Figure 2.

The CAD model is imported into ANSYS Workbench software, and the 3D hexahedral-dominant computational grid is generated from the channel geometry (Figure 2). The Sweep method is used to generate the mesh structure, in which the constant cross-section surface of the slide is discretized with quadrilateral and triangular elements using the Quad Dominant scheme and then swept through the body creating a volume mesh. Quadratic element order with the element size of 10 μm is considered. Mesh refinement is performed by analyzing the sensitivity of pressure drop and shear stress to the number of elements in the model, to ensure adequate discretization and the independence of the results to mesh size. More precisely, the analyses with coarser and finer meshes showed a negligible error when comparing results (relative error less than 5%). The selected mesh comprises 1,821,175 nodes and 407,560 elements, which is deemed to be adequate to guarantee a grid-independent solution.

The no-slip boundary condition is prescribed on all surfaces of the μ-slide channel except the inlet and outlets. Wall surfaces are assumed to be rigid. Furthermore, boundary conditions are set to a constant mass flow rate of 1.667 × 10^−7^ kg/s, equivalent to the volumetric flow rate of 0.01 mL/min, applied at the inlet section. This selected perfusion flow rate at the inlet is suitable for flow in a microfluidic device, as observed previously in [30]. At the outlets, a uniform normal stress field is set to be equal to the atmospheric pressure, hence static gauge pressure condition of 0 Pa is enforced.

The commercial CFD software ANSYS^®^ CFX Workbench (version 2022.R2, ANSYS Inc., Canonsburg, PA, USA) is used to carry out numerical simulations that determine the fluid dynamics and the produced fluid forces within the microfluidic device channel. In the CFX software, the porous media governing Equation (1) is formulated by adding a source term in the momentum equations. This momentum loss contributes to the pressure gradient in the porous cell using the permeability coefficient *K*_*p*_ to represent viscous losses, creating a pressure drop that is proportional to the fluid velocity in the cell. The ANSYS solver has the convergence criteria for residuals set to 10^−6^. The CFD simulations are performed to provide a prediction of the filtration velocity field and pressure gradient within the device channel, as well as local shear stress values. The produced numerical data and contour representations of these model predictions are discussed in Section 3.

## 3. Results

### 3.1. Fluid Dynamics Within _μ_-Slide: CFD Measurement of Velocity and Shear Stress

**Figure 3.**
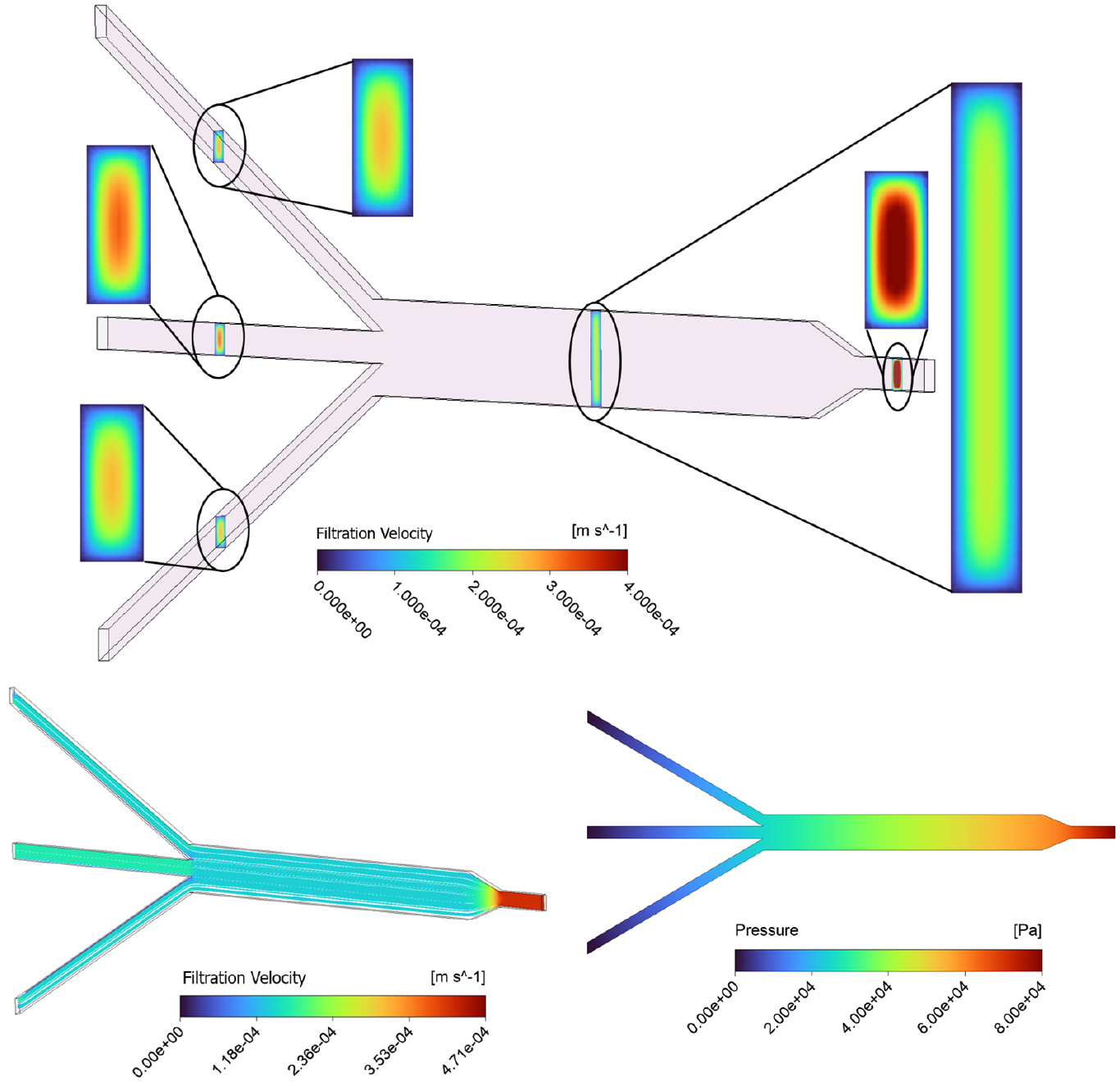
Contours of filtration velocity at five various rectangular cross-sections in the channel, including the 1 mm inlet, 3 mm main channel, and three 1 mm outlets for the baseline (**top**); filtration velocity streamlines (**bottom-left**), and a profile of pressure distribution along the porous domain for the baseline case (**bottom-right**).

The contour results of the baseline model (*K*_*p*_ = 10^−13^ m^2^) for filtration velocity and pressure distribution in the porous μ-slide are reported in Figure 3. The visualization of filtration velocity contours at five different cross-sections throughout the length of the channel is provided in the top panel of Figure 3. The slices are located at the 1 mm inlet, 3 mm main channel, and the three 1 mm outlet sections. The filtration velocity magnitudes are differentiated by the color bar. The “region of interest” for this study is the 3 mm section of the channel where cellular proliferation occurs under experimental conditions. The contour plots show that the highest filtration velocity occurs in the 1 mm inlet channel, with the flow in the 3 mm channel becoming decelerated due to the enlarged width of the slide and mass conservation. Similarly, the velocity slightly increases again in the 1 mm outlet channels. The analysis of the velocity map demonstrates that throughout the μ-slide, including the 3 mm channel, the filtration velocity profiles display a parabolic-like shape with a maximum velocity occurring in the center of the channel and a minimum velocity observed at the walls, due to the non-slip boundary condition applied to the surfaces of the device. We observe that along the larger dimension (i.e., width), the velocity shows a plateau in the center before it changes near the wall.

The fluid flow patterns within the μ-slide are evaluated using a streamline plot in the bottom-left panel of Figure 3. The visualization of streamlines is provided on a plane that cuts the μ-slide in half. The pattern of streamlines indicates the distribution of the flow within the channel, with the fluid moving parallel to the walls. The streamline plot further confirms that the velocity reaches the highest magnitude near the 1 mm inlet and outlet channels. Moreover, a uniform distribution of the filtration velocity with a constant value of 1.4 × 10^−4^ m/s in the main 3 mm channel region is observed. The fluid pressure contour of the device, pictured in the bottom-right panel of Figure 3, identifies a pressure decrease in the direction of flow throughout the channel, from the inlet to the outlets, with the highest pressure observed at the inlet and zero pressure defined at the outlets. The fluid in the porous scaffold is driven by this pressure gradient. The pressure field is shown on a plane that cuts the domain in half.

Additionally, wall shear stress (*WSS*) is evaluated, which is a mechanical stimulus generated by the friction of the fluid flowing in the μ-slide against the channel walls. The WSS is defined by Equation (2) as the product of fluid density and near-wall velocity gradient, where *h* denotes the x-, y-, and z-directions.

(2) $$WSS=\mu_{f}\frac{\partial v_{f}}{\partial h}$$

The results of the shear stress analysis are illustrated in Sections 3.1.1 and 3.1.2. In this context, the sensitivity of the model predictions to physical parameters such as hydrogel scaffold permeability and perfusion flow rate is explored. To analyze the influence of scaffold porous properties, the modeling approach is used to compare the fluid transport and evaluate the shear stresses in porous channels with different permeability values (Table 2). The results are also analyzed by adjusting the inlet flow rate to identify the relation with model predictions and parameters of the device.

#### 3.1.1. Effect of Matrix Permeability: Filtration Flow and Shear Stress Analysis

**Figure 4.**
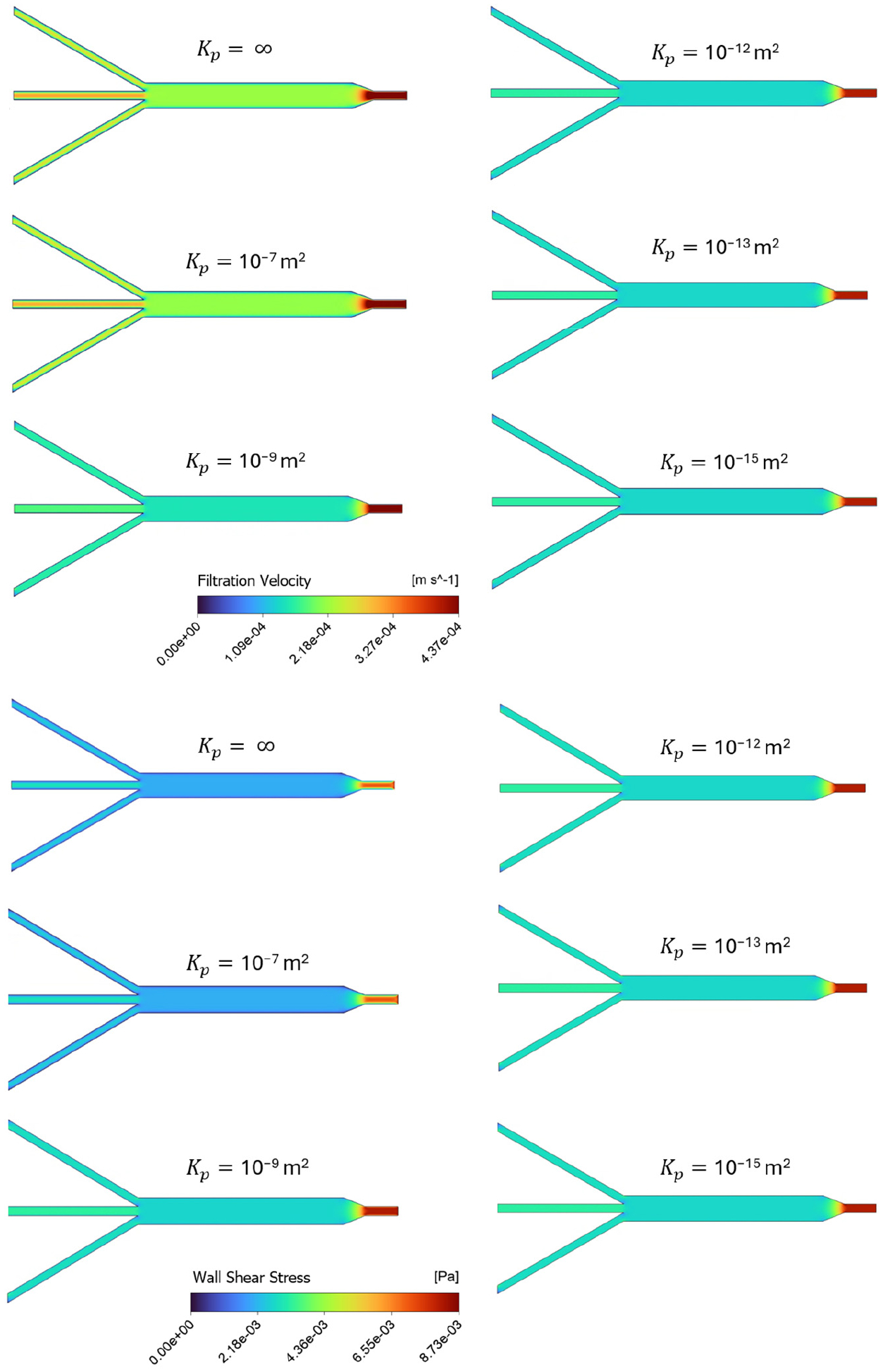
Channel profiles of velocity and shear stress for different simulation scenarios in Table 2, representing cases with varying permeability, on the same scale. The top panel shows the distribution of filtration velocity decreasing as permeability decreases, while the bottom panel presents the wall shear stress increasing as permeability decreases.

Figure 4 provides a series of screenshots depicting the contour plots of filtration velocities and shear stress distributions within the porous microslide for the cases with varying permeability, using an inlet flow rate of 0.01 mL/min. The six varying permeability cases in the range of *K*_*p*_ = 10^−15^ m^2^ to 10^−7^ m^2^ are listed in Table 2, where *K*_*p*_ = ∞ represents an infinite, permeable, non-porous fluid domain, and the case with the permeability value of *K*_*p*_ = 10^−13^ m^2^ is denoted as the baseline, chosen according to human ECM permeability in previous studies [24,31]. In the insets, velocity contours are plotted on a plane at the center of the channel due to geometrical symmetry, while the shear stress is displayed on a plane representing the channel wall boundary. Contour colors show the magnitude of the filtration flow and shear stress, and contour forms for all cases are on the same scale.

Overall, Figure 4 illustrates a uniform distribution of the filtration velocity and shear stress within the 3 mm channel. The shear stress in this section of the slide has a constant value of 2.8 × 10^−3^ Pa for the baseline case. The highest shear value is observed at the inlet region for all the cases, and as the width of the microchannels expands to the 3 mm section, the velocity and shear stress values tend to decrease. The contours in the top panel of Figure 4 show the filtration velocity decreasing as permeability decreases, with the most significant change observed in the highest permeability values (10^−7^ and 10^−9^ m^2^), while a relatively constant magnitude value is observed in the proceeding cases. The wall shear stress contours in the bottom panel of Figure 4 also present a more significant change in the highly permeable cases, but with shear stress increasing as permeability decreases. This means that the less permeable cases present constant, but higher shear values than the more permeable cases. Therefore, the model predicts an increase in shear stress and a decrease in filtration velocity when the channel permeability is decreased.

**Figure 5.**
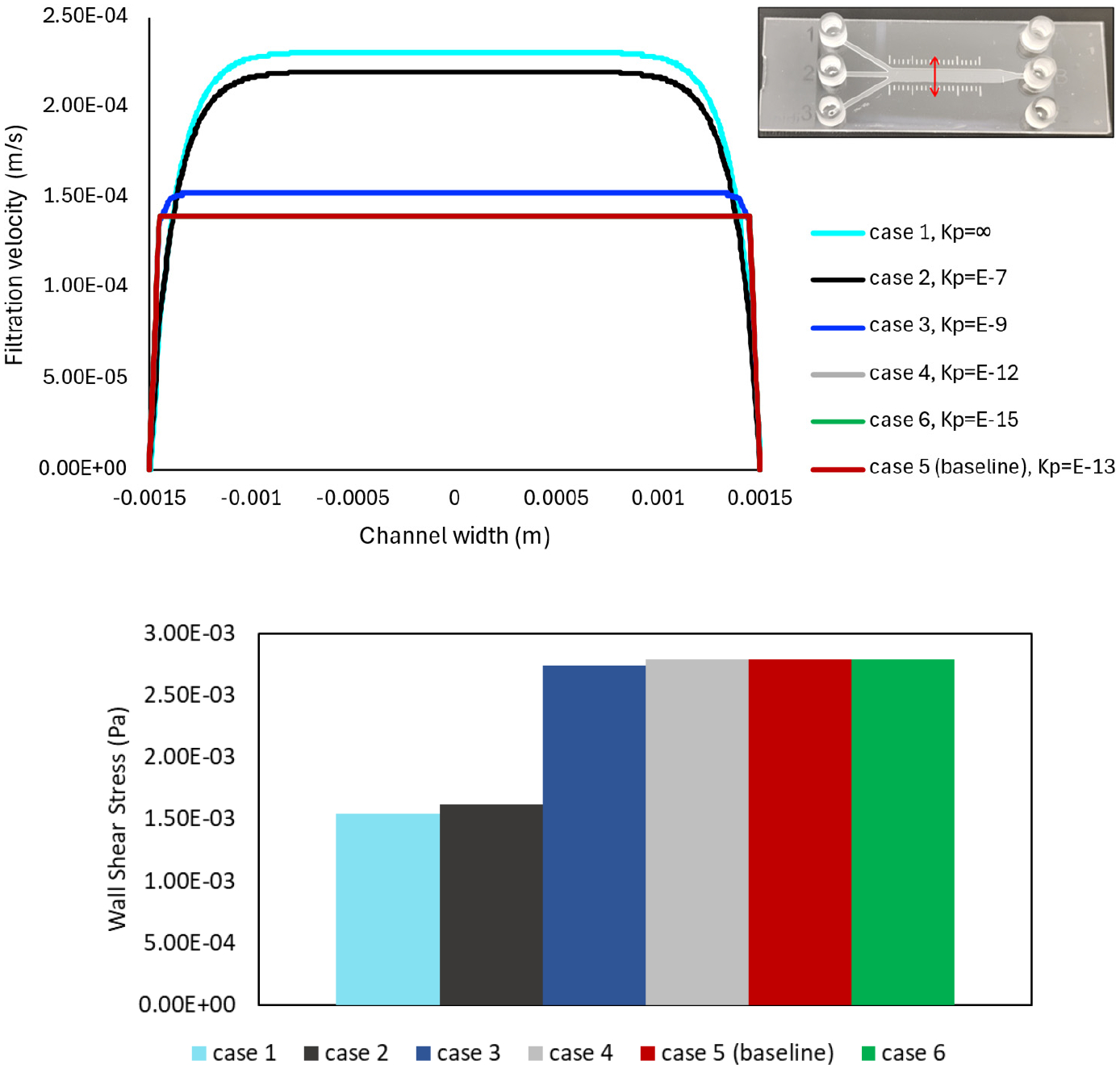
Filtration velocity and wall shear stress plots for the analyzed cases in Table 2, with six varying permeability values. The same cases are shown in Figure 4. The results are obtained on a cross-sectioned line at the middle of the 3 mm section along the channel width, shown in the top-right corner. The filtration velocity is presented by the line plot in the top panel, while the variation in shear stress value is represented by the bar chart in the bottom panel. No significant change in magnitude values is observed in either plot among the three lowest permeability value cases.

Furthermore, to quantitatively analyze the distribution of fluid within the microchannel, the filtration velocity profiles are plotted against the width of the 3 mm channel, and the wall shear stress values in the same region are compared in Figure 5, for six varying ECM permeability cases noted in Table 2. The path line is located at the center of the 3 mm section, capturing the region where the fully developed flow condition is achieved. A red double-headed arrow is used to represent the line in the geometry, shown in the top-right corner of Figure 5.

The top panel of Figure 5 displays the highest filtration velocity value in the highest permeability (fluid) case, and the velocity decreases as the permeability also decreases, in agreement with the contours in Figure 4. It can be noted that the lines for Cases 4 and 6, with *K*_*p*_ = 10^−12^ and 10^−15^ m^2^ (gray and green), are hidden behind the baseline line (red), presenting no significant change in velocity magnitude values across the channel between the low permeability cases. Additionally, as the permeability of the porous structure decreases, the velocity profile tends to flatten in the center of the microchannel, showing a plateau region. The filtration velocity profile has its maximum value in this plateau zone and decreases towards the walls of the device.

Additionally, the bar chart in the bottom panel of Figure 5 highlights the wall shear stress values following a similar behavior along the cases, but with shear values increasing as permeability decreases. Again, the magnitude values do not significantly change for the three lowest permeability cases (including the baseline) but present significantly lower shear values in the more permeable cases. Namely, given that wall shear stress changes linearly with the velocity gradient (Equation (2)), the maximum shear is found to occur in cases with lower permeability values.

#### 3.1.2. Effect of Perfusion Flow Rate: Filtration Flow and Shear Stress Analysis

The flow rate of the perfusion media varies depending on the research application and needs to be chosen very carefully, since shear force caused by flow can strongly impact the cells’ intrinsic physiology. Therefore, five different values of flow rate (*Q*_*in*_ = 10^−3^, 5 × 10^−3^, 10^−2^, 5 × 10^−2^, and 10^−1^ mL/min) are applied at the inlet of the porous baseline case (*K*_*p*_ = 10^−13^ m^2^), and the results are compared to evaluate their effects on shear stress and velocity profile distribution within the channel, respectively.

**Figure 6.**
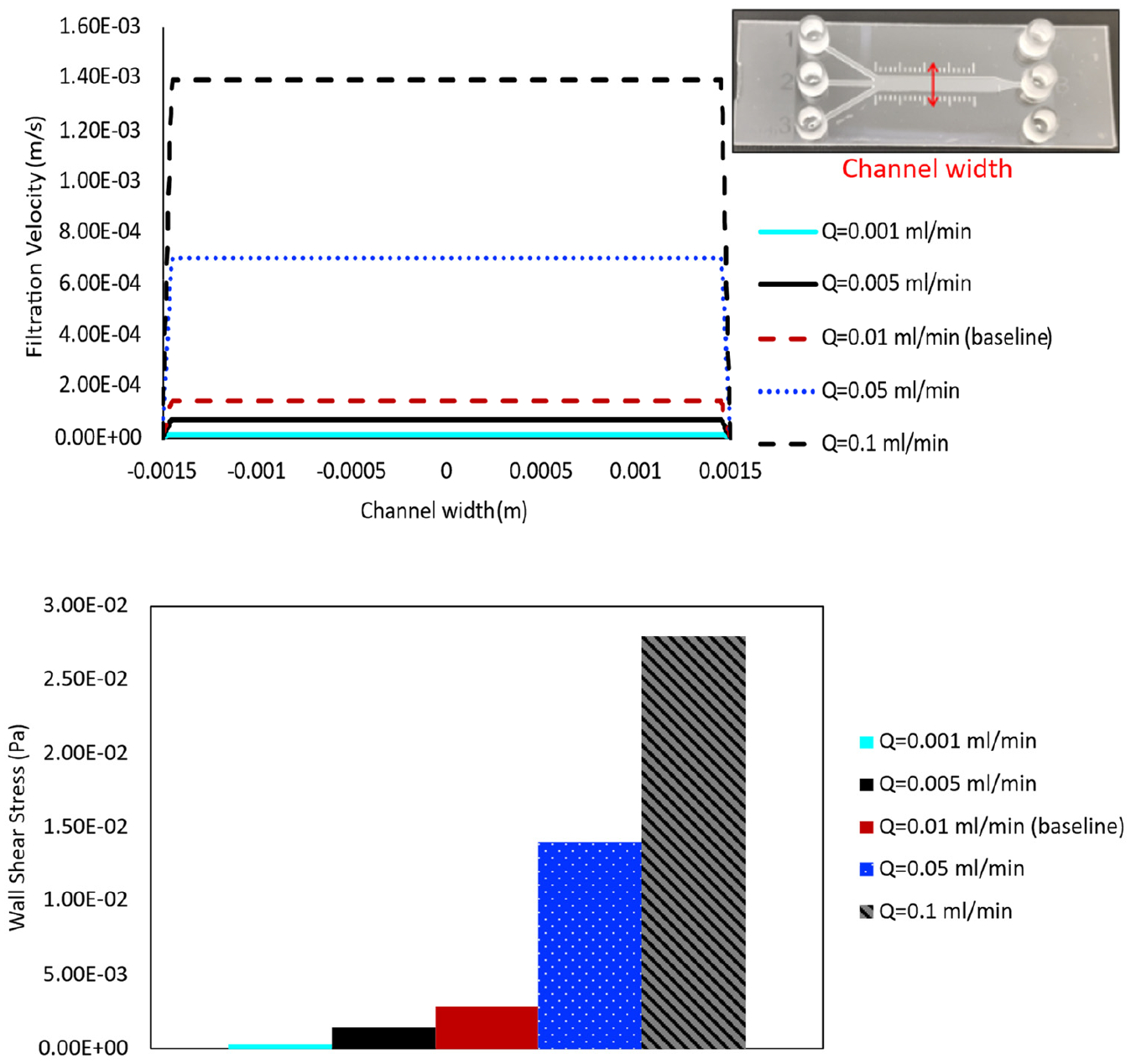
Filtration velocity profiles and shear stress value at five different inlet flow rates are compared for the case with *K*_*p*_ = 10^−13^ m^2^. In addition to 0.01 mL/min (baseline case), flow rates of 0.001 mL/min, 0.005 mL/min, 0.05 mL/min, and 0.1 mL/min are simulated. The results are obtained in a cross-sectional line along the channel width, shown in the top-right corner. The filtration velocity is presented by the line plot (**top panel**), while the shear is represented by the bar chart (**bottom panel**).

The results quantifying filtration velocity profiles and wall shear stress values for cases with varying inlet flow rate are shown in the top and bottom panels of Figure 6, respectively. The velocity profile is obtained along the line across the channel width, halfway along the length of the main 3 mm channel, shown in the top-right corner of this figure. Comparing the velocity over this line for cases with different medium flow rates illustrates the highest filtration velocity value in the highest inlet flow rate case (0.1 mL/min), and velocity decreases as flow rate decreases (Figure 6, top panel). The most significant changes are observed between the highest flow rate cases, with larger jumps occurring from approximately 1.4 × 10^−3^ m/s (*Q*_*in*_ = 0.1 mL/min) to 7.0 × 10^−4^ m/s (*Q*_*in*_ = 0.05 mL/min) and 1.4 × 10^−4^ m/s (baseline case). The lowest flow rate cases show consistently decreasing values, but with a smaller magnitude of change due to their lower overall values, as seen in 7.0 × 10^−5^ m/s and 1.4 × 10^−5^ m/s for inlet flow rates of *Q*_*in*_ = 0.005 and 0.001 mL/min, respectively. The bar chart in the bottom panel of Figure 6 highlights the wall shear stress values that follow a similar trend across the cases, with shear increasing as flow rate increases. The more noticeable changes occur among the highest flow rate cases, although consistent differences are also observed among the lowest flow rate cases.

### 3.2. Measuring Filtration Velocity In Vitro

**Figure 7.**
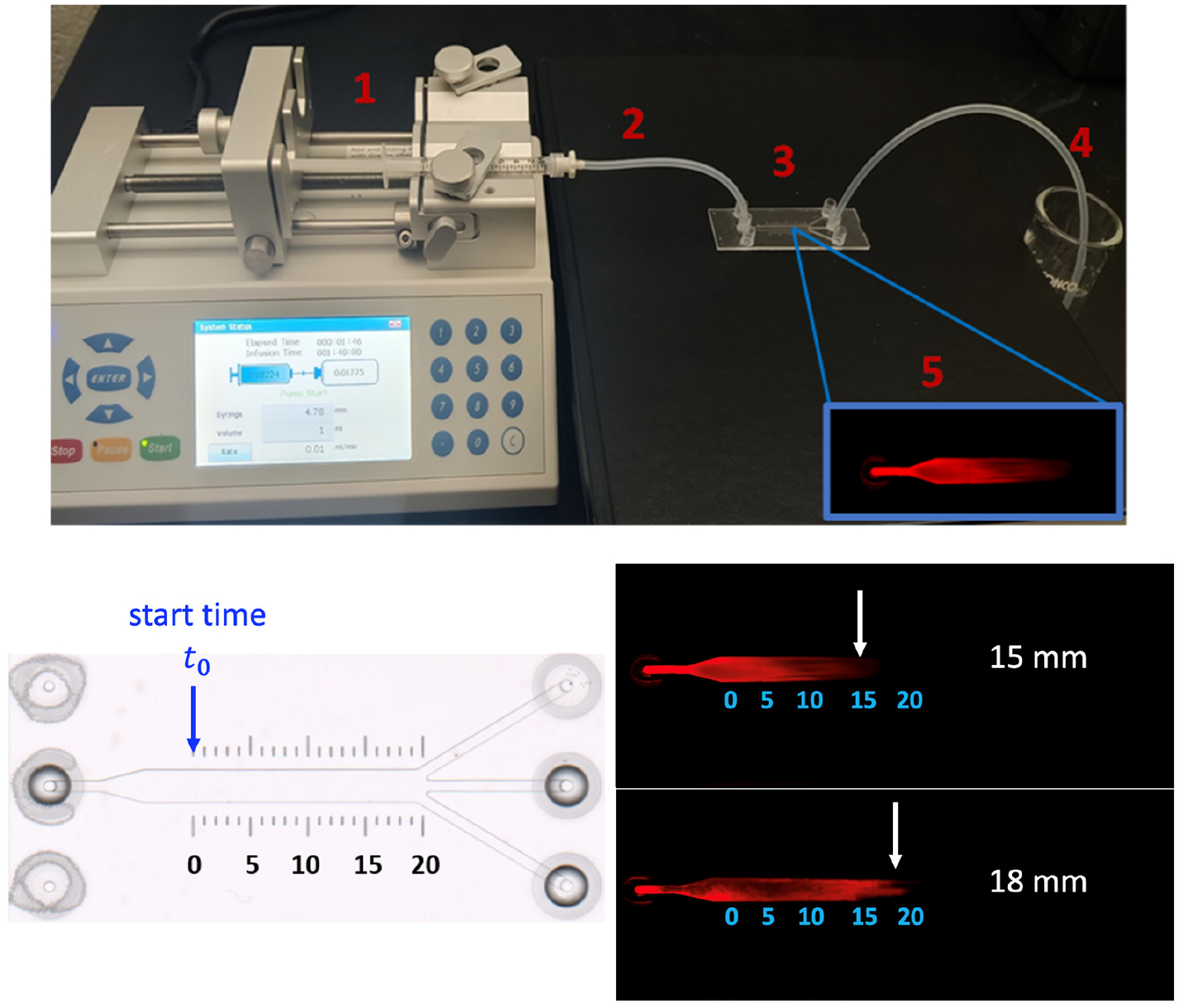
Illustration of overall experiment procedure for filtration velocity measurement in the μ-slide (**top panel**), including the preparation of the system (1–4) and imaging process under a microscope (5). (**bottom panel**) imaging of ECM filled micro-slide prior to SA-680 diffusion (**left**) and the displacement of SA-680 across the HumaMatrix-filled ibidi μ-slide (**right**). Two experiments (n = 2) are performed with the same inlet flow rate. For the calculation of velocity, the starting time is noted after SA-680 has moved in the channel to 0 mm point.

In this section, we determine filtration velocity via experimental conditions. A saline-based buffer containing a dye-conjugated small protein that is continuously perfused through a natural ECM is used to represent fluid flow in the porous tissue structure. A flow rate of 0.01 mL/min is used at the inlet to mimic convection in the interstitial space. The matrix and buffer are confined to a commercially available microfluidic channel with a defined dimension and optimized for optical and fluorescence imaging. The filtration velocity is calculated using the average time it takes for the protein molecules to pass through the channel. The experimental setup for visualizing flow perfusion in the ECM-embedded μ-slide is shown in the top panel of Figure 7. The velocities predicted by the CFD numerical model are compared with experimental measurements.

#### 3.2.1. Preparation Process and Details

The same device used for the CFD simulations, ibidi μ-slide III 3-in-1 is utilized for the experiments, which consists of a channel with defined geometrical dimensions summarized in Table 1. The scale bar printed on the slide is used for distance measurement. The ibidi slides are purchased from ibidi GmbH (Germany). HumaMatrix^®^ extracted from nutrient-rich human birth tissue, is used as an ECM-based porous biomaterial filled in the slide. This native human-derived ECM contains matrix proteins and growth factors, and has been used for hydrogel formation and modifying surfaces for in vitro cell attachment [32]. HumaMatrix^®^ is purchased from Thermo Fisher Scientific (Hampton, NH, USA). Streptavidin IRDye 680 (SA-680), obtained from Li-COR, Inc. (Lincoln, NE, USA), is used for tracking the fluid flow in the channel.

A programmable syringe pump (Fusion 200) is used to perfuse SA-680 unidirectionally at a precise and constant flow rate into the channel. The pump is equipped with a syringe holder, and it is connected to a silicone tube. The setup is pictured in the top panel of Figure 7 and consists of the following components: integration of Fusion 200 pump with the syringe (1), attachment of silicone tubing connecting the syringe to ibidi slide (2), microfluidic ibidi slide filled with HumaMatrix to facilitate controlled perfusion of SA-680 (3), collection of waste effluent from the slide (4), and finally the imaging of perfused dye with Odyssey system (5) for velocity analysis.

#### 3.2.2. Experimental Observation and Imaging: Velocity Measurement

The μ-slide channel is filled with the 95 μL matrix (3.3 mg/mL diluted in phosphate-buffered saline (PBS)) and allowed to equilibrate for 2 h at room temperature. It should be noted that while the channel volume is 60 μL, the fluid volume of 95 μL is introduced to fill voids in the entire passage to avoid air pockets. A syringe pump connected to a 1 mL syringe containing 0.1 mg/mL of SA-680 is used to perfuse the solution into the matrix-filled channel over 2 min.

The velocity of SA-680 moved through the channel is estimated based on the distance traveled over the period, with the start time *t*_0_ defined when SA-680 is at the 0 mm point of the main channel length (Figure 7). The detection of SA-680 diffusion across the matrix-filled channel is imaged using a Li-COR Odyssey imager at a resolution of 100 microns. The experiment is repeated twice with two separate slides (n = 2). The analysis of the fluorescent images showed migration of SA-680 across the matrix-filled channel driven by a constant flow rate (0.01 mL/min) as shown in the bottom panel of Figure 7. Filtration velocity values of 2.14 × 10^−4^ and 2.25 × 10^−4^ m/s are determined by calculating the distance traveled by the protein in each slide (n = 2, 15 mm and 18 mm), divided by the corresponding time intervals. These results demonstrate a method for measuring the velocity of small globular proteins moving through a biological matrix under constant convective flow. The method is optimized to the extent that the movement of SA-680 is sensitive to matrix filling and air pockets. Additionally, the perfusion rate is selected to create a laminar flow of the protein since the interstitial fluid flow in tissues and microvasculature ranges from 0.01 to 0.1 mL/min [33]. It should be noted that localized regions of high and low porosity are expected in the channel filled with native ECM, which can result in voids in the channel flow (Figure 7, bottom panel). Human ECM is primarily composed of collagen, fibronectin, laminin, and glycoproteins, and each component would interact with the device surface differently. The apparent void could cause the solution to deviate from parabolic flow. These effects have been reported in the literature that discusses heterogeneous porosity in ECM-based materials and its impact on fluid flow [32,34].

**Figure 8.**
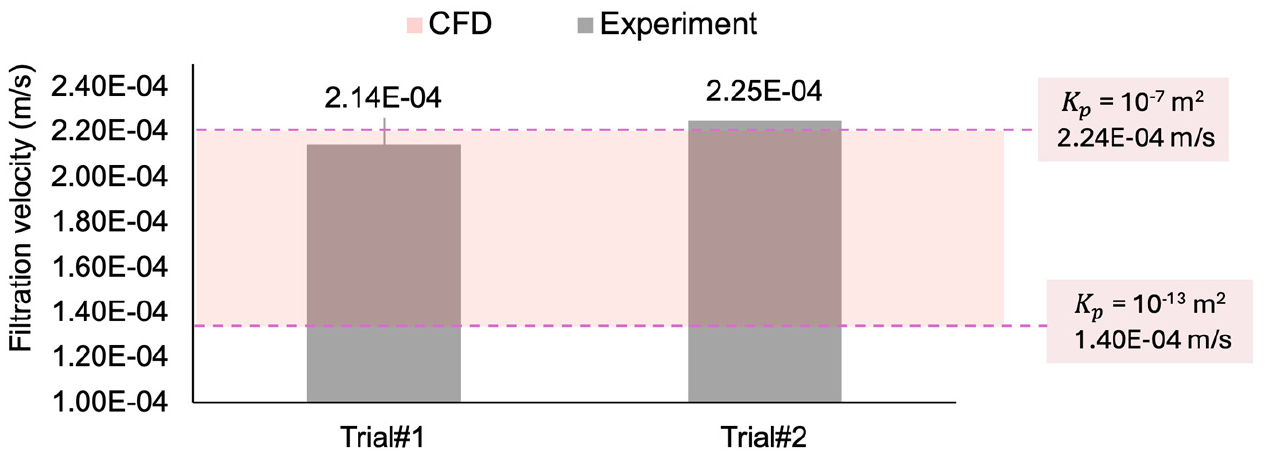
Comparing the filtration velocity between two experimental trials and the range of CFD simulation results.

Figure 8 compares the experimental filtration velocity results from the two in vitro trials (Figure 7) with the values calculated from the CFD simulations. The in vitro experimental measurements (2.14 × 10^−4^ and 2.25 × 10^−4^ m/s) are in the range of the computational values (between 1.4 × 10^−4^ m/s and 2.24 × 10^−4^ m/s, for the permeability coefficients of 10^−15^ to 10^−7^ m^2^, respectively), suggesting good agreement between the computational and experimental data. While only two experimental trials are conducted, the consistency of the CFD result within the observed range supports its reliability as a method for simulating this study’s in vitro flow conditions.

### 3.3. Validation with Theoretical Calculations

This section presents validation of the CFD model using a fluid-only channel. The microfluidics’ μ-Slide III 3in1 (ibid Inc.) Application Note [35], containing the equations for the theoretical model, is used to further verify the computational simulations, particularly the computed velocity and shear stress values within the channel.

**Figure 9.**
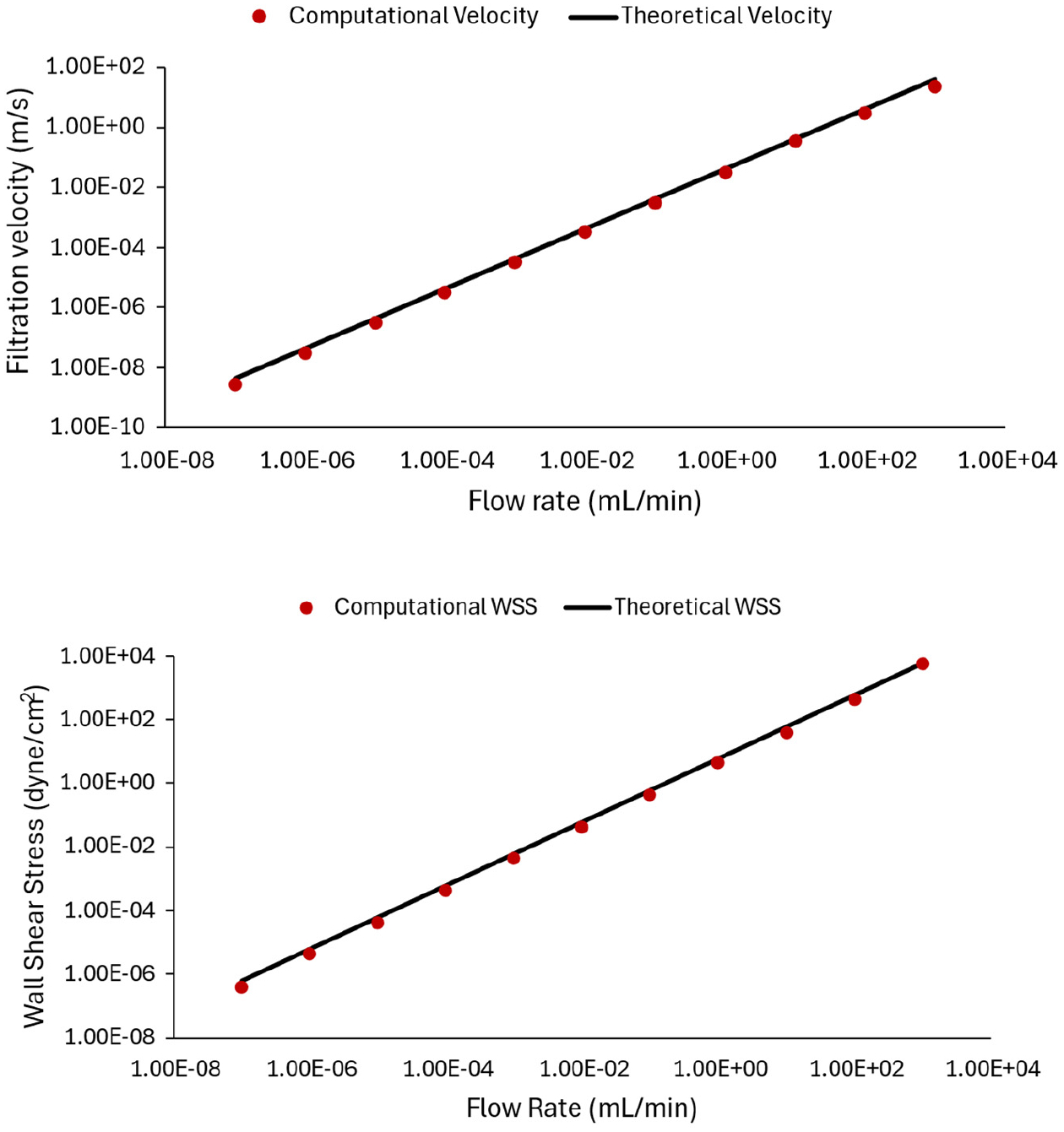
Comparing CFD results and theoretical data: plots illustrating the relationship between filtration velocity (**top panel**) and wall shear stress (**bottom panel**) with inlet flow rate. The computational data points from ANSYS CFX are represented by red circles, while the theoretical values computed by Equation (3) are shown by the black line. We observed that the simulation results are very close to theoretical values.

Figure 9 compares the computational results from ANSYS CFX with the corresponding theoretical results, plotted against varying flow rates (*Q*_*in*_). The relationship between flow rate and wall shear stress obtained from theory (black line) and CFD model data (red circular markers) is shown in Figure 9. Namely, to obtain computational data points for Figure 9, the flow rate condition at the inlet is varied from 10^−7^ mL/min to 10^3^ mL/min, and the CFD model is solved for each case to provide a prediction of the velocity and shear stress inside the channel. Subsequently, the flow-induced wall shear stress value and velocity profile inside the rectangular channel are calculated using Equation (3b) for filtration velocity and (3a) for WSS.

(3a) $$WSS=227.4\;\mu_{f}Q$$

(3b) $$V_{f}=\frac{Q}{A}$$

In Equation (3), *μ*_*f*_ is the fluid medium dynamic viscosity in dyne.s/cm^2^, *Q* is the channel volumetric flow rate in mL/min, and *A* is the cross-sectional area of the slide in m^2^, calculated using the provided channel width and height in Table 1. The constant of 227.4 in this formula includes the unit conversions to represent the WSS in dyne/cm^2^. Additionally, *V*_*f*_ represents the average flow velocity in the channel. It should be noted that the theoretical values are estimated at 3 mm channel. Also, the theoretical velocities obtained from (3b) represent the average velocity and are multiplied by two to obtain the maximum velocity in the middle of the slide.

In the top panel of Figure 9, theoretical velocity values are computed for the chosen flow rate conditions and are compared with CFD predictions. The bottom panel of Figure 9 represents cases where the flow rate is varied, and shear stress values are compared. The results in Figure 9 demonstrate a strong match between the graphical representations, expressing that the theoretical calculations matched computational results, and indicating the accuracy and reliability of the computational simulations. The results also demonstrate how different flow rates affect shear stress and velocity within a microfluidic device.

**Figure 10.**
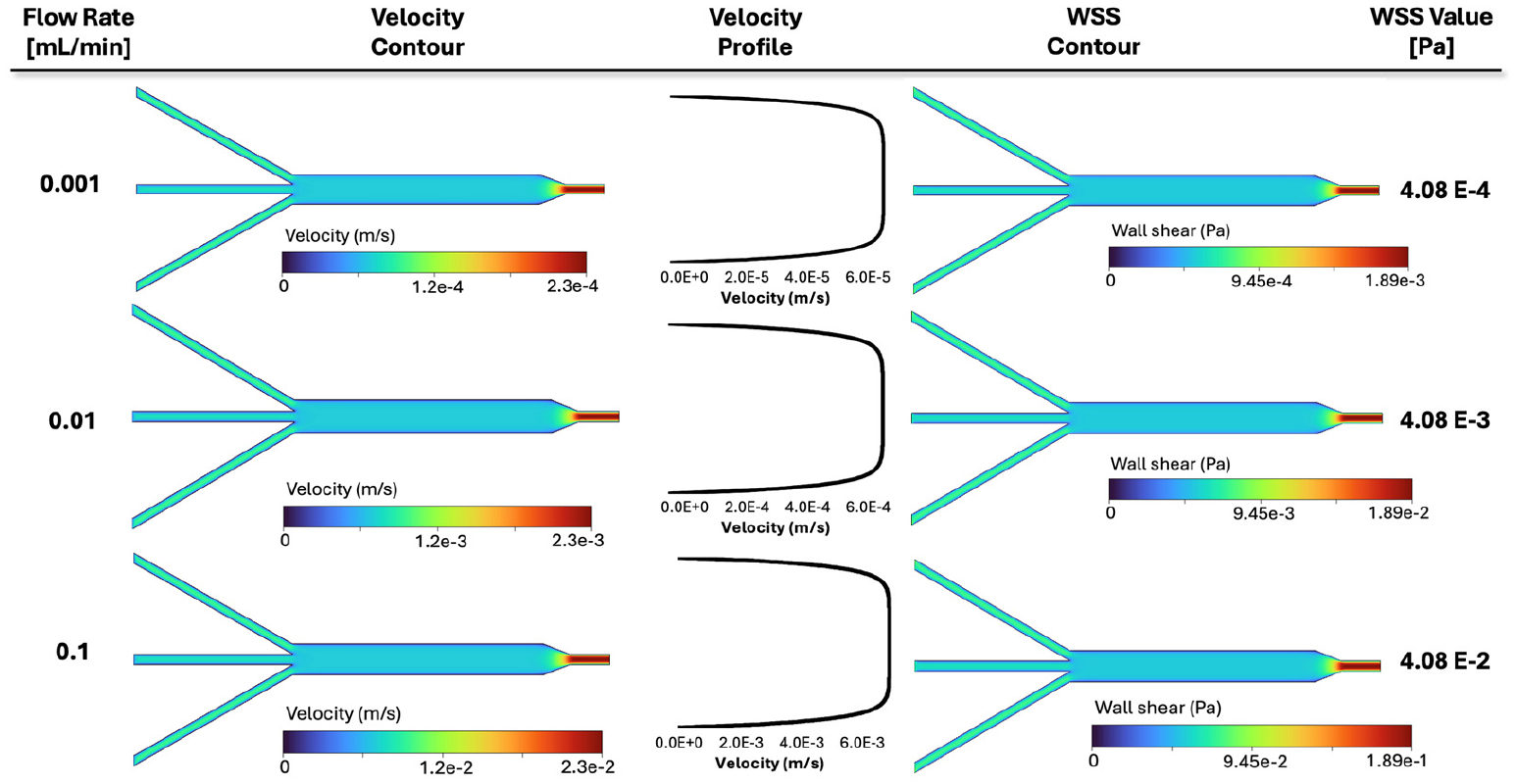
Simulation results for three representative cases in Figure 9 with varying inlet flow rates: velocity contours, velocity profiles, WSS contour, and WSS value in the 3 mm section are illustrated, respectively. The velocity profiles are plotted along a line in the middle of the channel, similar to Figure 5. Each contour is displayed with its own color bar, highlighting the effect of the inlet flow rate in fluid dynamics within the slide.

Consequently, the CFD contours for three representative cases of Figure 9 are plotted in Figure 10, demonstrating how various inlet flow rates affect fluid dynamics. Namely, from top to bottom, the results related to different flow rates of 10^−3^ mL/min, 10^−2^ mL/min (baseline, shown in Table 2), and 10^−1^ mL/min are displayed. From left to right, the velocity contours and velocity profiles versus the width of the 3 mm channel, as well as the resultant shear stress contour and its corresponding value in the 3 mm channel, are presented in Figure 10. Three rows are related to the above three flow rates. The middle contour reflects the baseline flow rate (0.01 mL/min) investigated previously in Figure 5, while the top and bottom represent that value divided and multiplied by a factor of 10, respectively. Individual color bars are used to display the contours, highlighting the drastic change in filtration velocity and wall shear stress between the cases. As the flow rate increases, both velocity and shear also increase by a factor of 10. The same happens with the plotted velocity profiles, while the lines still have a similar slope. It can be observed that the fluid spread throughout the chamber assumes a different velocity magnitude depending on the applied flow rate at the inlet of the device. Also, the shear stress scales linearly with flow.

## 4. Discussion

In this work, a CFD framework is employed to predict filtration flow and the corresponding local shear stress fields within a μ-slide channel commonly used in bioartificial organ engineering. The flow is driven by the moving of an aqueous fluid through a biological matrix. The model includes a porous component representing the scaffold, which characterizes fluid flow behavior through natural human ECM.

The filtration velocity is also determined based on the movement of a dye-conjugated small globular protein tracked using a near-infrared imaging system. The results of the computer solver are consistent with experimental observations (Figure 8). Moreover, the sensitivity of the model predictions to physical parameters such as inlet perfusion flow rate and matrix permeability is explored. The porous modeling in ANSYS CFX has been previously validated in various applications, such as vocal fold tissue [36,37], and cardiovascular biomechanics [38,39]. Furthermore, shear stress and velocity for the flow rate provided by the syringe pump are calculated based on the equations in the μ-slide Application Note [35], which provides the same values as the calculation from CFD model predictions. The variation in the slide shear stress and velocity with respect to the flow rate in Figure 9 (theory and simulation compared) confirms these consistent observations.

The filtration velocity profiles across the porous microchannel, the resultant wall shear stress contours, and values of these variables are presented and summarized in Figures 4–6, demonstrating how various simulation physical properties affect fluid dynamics. A large area of uniform shear stress and homogeneous velocity distribution through the channel is observed (Figure 4), which has been suggested to promote cell adhesion and cell alignment in the fluid direction [40], hence ensuring cell survival. However, changes in porous structure properties can cause changes in fluid transport. The simulations result in Figure 5 for the cases with six varying matrix permeability values demonstrate that for higher permeability values, the filtration velocity increases, and the shear stress value decreases. Conversely, the shear stress increases for cases with smaller values of permeability coefficients (Figure 5), while the relative velocity between the fluid and solid phases (filtration velocity) decreases. Specifically, as permeability decreases from Case 1 to 6, lower velocity values and higher shear stress values are observed, in agreement with data in [19]. This is because the near-wall velocity gradient is higher in the porous microchannel compared to the fluid-only (non-porous) microchannel. Nevertheless, there are no differences between the shear stress and filtration velocity profiles in cases with the permeability lower than the baseline value of *K*_*p*_ = 10^−13^ m^2^. Figure 5 helps us to evaluate the role of ECM permeability in the fluid transport behavior within the slide. More precisely, simulations demonstrate that in the 3 mm channel, while a smaller permeability results in a lower filtration velocity, the velocity is similar for all the cases with permeability lower than 10^−12^ m^2^. The results indicate that ECM permeability below this value does not affect the filtration velocity and wall shear stress pattern in a sensitive manner. The shear slightly decreases by increasing the permeability to *K*_*p*_ = 10^−9^ m^2^.

Furthermore, the filtration velocity and wall shear stress indicated different values at different flow rates, as expected, with both parameters appearing to increase as the inlet flow rate increases (Figure 6). Namely, increased fluid flow also led to an increase in wall shear stress, which has the potential for deformation or erosion. Therefore, the shear stress in a rectangular channel is dependent on the flow rate of the perfused medium.

It is found that the filtration velocity profile is directly proportional to permeability and directly proportional to the flow rate within the microfluidic channels. It is also found that the permeability of the ECM-embedded channel and inlet perfusion rate could affect filtration velocity profiles, contributing to the control of shear stress. Overall, as permeability increases, the velocity in the porous channel decreases, and the shear stress is proportionally affected. Therefore, enhanced permeation combined with lower inlet flow rates causes a decrease in the shear level within the chamber.

This methodological approach provides a framework for investigating the effects of shear stress in a porous medium situated in a microfluidic device, contributing to the optimization of conditions for tissue engineering, drug testing, and disease modeling. The findings provide insights into the interaction between fluid and porous constructs, which are essential for ensuring accurate simulation of the microfluidic environment, as well as optimizing flow perfusion operating conditions to manage cell proliferation. Future studies could further refine the model by capturing the critical shear stress range and analyzing the impact of fluid flow parameters in predicting viable operating space for different ECM properties.

## 5. Conclusions

We numerically investigated the fluid dynamics parameters within a porous matrix in a prefabricated microfluidic device and presented the validation of CFD predictions using theoretical calculations. Additionally, the filtration velocity obtained from the computational approach is of the same order of magnitude as the value obtained in the in vitro experiment. The simulations suggest that the scaffold permeability coefficient and inlet flow rate play a relevant role in fluid transport and wall shear stress values, where lower permeability and higher inlet flow rates caused shear values to increase. We conclude that the simulations provide a valuable insight into the wall shear stress distribution within the μ-slides.

## Figures and Tables

**Table 1.** Geometrical and physical parameters for the slide domain and their values.

Domain	Parameter	Value
Slide channel geometry (μ-Slide III 3in1)	Outer width *W*	25.5 mm
	Outer length *L*	75.5 mm
	Channel length *l*_*f*_	45 mm
	Total channel volume	60 μL
	Channel height *H*	0.4 mm
	Width of channels thin/thick	1 mm/3 mm
Culture medium fluid (water)	Density *ρ*_*f*_ Dynamic viscosity *μ*_*f*_	998 kg/m^3^ 0.001 kg/m.s
Porous matrix (human ECM)	Permeability *K*_*p*_ Porosity *γ*_*p*_	1 × 10^−13^ m^2^ (baseline) Refer to Table 2 for range of variation 90%
Boundary conditions	Fluid inlet flow rate *Q*_*in*_ Fluid outlet pressure *p*_*out*_	0.01 mL/min (baseline) equal to 1.67 × 10^−7^ kg/s 0 Pa

**Table 2.** Parameter sets for simulated cases. The different values for permeability are listed for Cases 1 to 6. Case 1 represents the situation when *K*_*p*_ → ∞; Case 5 is defined as the baseline.

	Case 1 (Non-Porous)	Case 2	Case 3	Case 4	Case 5 (Baseline)	Case 6
*K*_*p*_ (m^2^)	∞ (fluid only)	1 × 10^−7^	1 × 10^−9^	1 × 10^−12^	1 × 10^−13^	1 × 10^−15^

## Data Availability

The original contributions presented in the study are included in the article, further inquiries can be directed to the corresponding author.
